# By Reverence, Not Fear: Prestige, Religion, and Autonomic Regulation in the Evolution of Cooperation

**DOI:** 10.3389/fpsyg.2019.02750

**Published:** 2019-12-17

**Authors:** Hillary L. Lenfesty, Thomas J. H. Morgan

**Affiliations:** ^1^School of Human Evolution and Social Change, Arizona State University, Tempe, AZ, United States; ^2^Institute of Human Origins, Arizona State University, Tempe, AZ, United States

**Keywords:** autonomic nervous system, evolution of cooperation, prestige, dominance, evolution of religion, cultural evolution

## Abstract

Recent evolutionary theories of religions emphasize their function as mechanisms for increasing prosociality. In particular, they claim that fear of supernatural punishment can be adaptive when it can compensate for humans’ inability to monitor behavior and mete out punishment in large groups, as well when it can inhibit individuals’ impulses for defection. Nonetheless, while fear of punishment may inhibit some anti-social behaviors like cheating, it is unlikely to motivate other prosocial behaviors, like helping. This is because human physiology has evolved separate neurological systems with differential behavioral correlates either for (1) processing fear and responding to threats or (2) facilitating social interactions in environments which are deemed safe. Almost all vertebrates possess autonomic pathways for processing threats and fear, which result in “fight,” “flight,” or “freeze” responses and so likely mediate interactions in dominance hierarchies. Mammals, however, possess an additional, phylogenetically newer, pathway dedicated to suppressing such defensive responses in the service of promoting social affiliation or engagement. Here, we argue that this mammalian physiology supports an alternative hierarchical system unique to humans: prestige. In contrast to dominance, which involves aversion, fear and shame, prestige hierarchies are characterized by physical proximity and eye-contact, as well as emotions like admiration and respect for leaders. Prestige also directs the flow of cultural information between individuals and has been argued to have evolved in order to help individuals acquire high quality information. Here, we argue that not only does the mammalian autonomic pathway support prestige hierarchies, but that coupled with prestige biased social learning, it opens up a means for prestigious figures, including deities, to support the spread of prosocial behaviors. Thus, in addition to theories that emphasizes religious fear as a motivating factor in the evolution of prosocial religions, we suggest that reverence – which includes awe and respect for, deference to, admiration of, and a desire to please a deity or supernatural agent – is likely just as important. In support of this, we identify cases of religions that appear to be defined predominantly by prestige dynamics, and not fear of supernatural punishment.

*Awe, unlike fear, does not make us shrink from the awe-inspiring object, but, on the contrary, draws us near to it*.

(Heschel, 1976)

Be imitators of me, as I am of Christ.

-1 Corinthians 11:1:

The Guru is the conveyance in which the spiritual influence is brought to you. Anyone can teach, but the spirit must be passed on by the Guru to the Shishya (disciple), and that will fructify.

(The Complete Works of Swami Vivekananda, 1895)

## General Introduction

In recent years, evolutionary explanations of religion have proposed that omniscient, moralizing gods can facilitate cooperation by inducing fear among people and threatening them with punishment for their transgressions (Norenzayan, 2013; Johnson, 2015; Norenzayan et al., 2016; Purzycki et al., 2016). Indeed, gods have been and are feared, but historical accounts and contemporary religious practices show that supernatural agents (gods and saints) and their human representatives (such as priests, gurus, and martyrs) are also profoundly esteemed, adored, loved, respected, awed, freely deferred to, and imitated: in short, they are revered. Revered agents are also often described as being generous and prosocial. In turn, this may inspire a significant amount of generosity and prosociality among their followers without fear.

The goal of this manuscript is to advance a specific hypothesis about the evolution of human prosociality. By prosociality, we mean both norm-abiding behaviors and also costly cooperative behaviors like helping and sharing. Our hypothesis is that benevolent supernatural agents and their human representatives may be as effective at promoting prosociality as fearsome gods. This hypothesis is relevant to self-domestication because this path to prosociality is only possible in humans due to the evolution of prestige psychology in response to our constructed cultural environments. Prestige psychology itself may rely on the evolved structure of the mammalian autonomic nervous system (ANS). Furthermore, unique human modifications to the ANS compared with other mammals may have evolved in response to human cultural practices.

This paper is presented in four sections. First, we will outline the key differences between prestige and dominance hierarchies among animals and humans. Second, we will explain the structure and evolution of the mammalian ANS and how it influences social relationships, especially within prestige and dominance hierarchies. Third, we will discuss how the presence of gods and supernatural agents in both prestige and dominance hierarchies can lead to prosociality. Finally, we will present examples of prestige across religions as well as a focused case study of prestige-driven religious change in the first few hundred years of Christianity under the Roman empire.

## Dominance and Prestige

In dominance hierarchies, individuals often use their strength to threaten and coerce others and gain preferential access to mates and food. Among primates, relative rank is established between members of the same sex and collective awareness of the hierarchy shapes social behavior. Some adult male chimpanzees use intimidation and force to obtain and sustain their dominance status, including agonistic behavior like charging displays (Simpson, 1973; Bygott, 1979) and contact aggression (Watts, 2000a,b; De Waal, 2007), while other male chimps garner support from others over time by grooming them and in turn receive coalitionary support during physical fights in rank contests (Foster et al., 2009). In baboons, high-ranking females have access to the best feeding sites and can keep others away from these sites (Boyd and Silk, 2015) and higher-ranking females have been observed to take in 30% more food than lower-ranking females (Barton and Whiten, 1993).

Rank in dominance hierarchies is associated with fertility among several non-human primates including female chimpanzees, baboons, macaques, gray lemurs, marmosets, and tamarins (Dunbar and Dunbar, 1977; Dietz and Baker, 1993; Digby, 1995; Pusey et al., 1997; van Noordwijk and van Schaik, 1999; Radespiel and Zimmermann, 2001). This is true for other mammals as well: both high-ranking male and female hyenas have increased feeding time at carcasses; high-ranking females have more offspring who survive to maturity, and high-ranking males have more mating opportunities (Owens and Owens, 1996). Females are often aggressive with other females. Amongst naked mole-rats and meerkats, females will fight each other following a dominant breeder’s death to win her place (Reeve and Sherman, 1991; Clutton-Brock, 2007). When dominant female meerkats are pregnant, they will antagonize subordinate pregnant females and temporarily evict them from the group, and this stress may result in the subordinate’s abortion of the fetus (Young et al., 2006). In addition to mammals, social behavior in dominance hierarchies have been documented in social bees and wasps (Bourke, 1994), fish (Grosenick et al., 2007), and birds (Piper, 1997).

In humans, dominance dynamics include a range of avoidance behaviors. Low-status individuals avoid proximity to dominant individuals, diminish their posture in the presence of dominant individuals, avert their gaze (Henrich and Gil-White, 2001; Henrich, 2016), and raise their vocal pitch (Cheng et al., 2016). Subordinate or low-status individuals may sometimes mimic certain behaviors of dominant individuals, but this is argued to occur only to the extent required to satisfy the dominant and not in cases unmonitored by the dominant (Henrich, 2016). Dominant individuals have more erect posture (Weisfeld and Beresford, 1982) and lower vocal pitch (Cheng et al., 2016) than subordinates; they also are louder, talk more often, and interrupt others more frequently (Octigan and Niederman, 1979; Berger, 2008). Systems of dominance among humans are suggested to be associated with the emotions of shame, fear, and fear-based respect for the dominant, on behalf of low-status individuals (Henrich, 2016).

While dominance hierarchies exist among both humans and non-human animals, only humans possess an additional type of social hierarchy: prestige. Prestige hierarchies are characterized by qualitatively different emotions and behaviors than dominance hierarchies. A prestigious individual is someone who is esteemed for their skill, knowledge, or success in locally valued domains (Henrich et al., 2015). A few examples include: tool-making and tool-use; storytelling, medicine, cooking, and hunting; expertise in rituals, religion, etiquette and social norms; and acquiring wealth. Low-status individuals are likely to show direct gaze toward and desire to be near prestigious individuals, and they demonstrate “preferential, automatic, and unconscious” imitation of them (Henrich and Gil-White, 2001; Henrich, 2016). Followers/copiers (lower-status individuals) feel admiration, awe^1^, and respect – reverence – for the prestigious figure.

Prestigious figures are well-liked compared to dominant individuals who are not liked (Cheng et al., 2013). Rather than using fear-inducing threats to coerce others, prestigious figures gain influence simply by being good at something and valued by others for it; moreover, prestigious individuals show generosity and benevolence toward low status individuals (Radcliffe-Brown, 1964; Henrich and Gil-White, 2001; Henrich, 2016). In exchange for their expertise and generosity, prestigious figures receive “freely conferred deference” from low-status individuals (Henrich and Gil-White, 2001). This means that leaders don’t command deference by force, and that followers don’t show deference simply to reduce the threat from the leader; rather, followers willingly defer to the leader.

Although evidence for prestige outside our species is virtually non-existent, based on ethnographic observations, prestige dynamics appear to be widespread across human societies. For instance, they have been documented in small-scale, mobile societies in the domains of hunting, shamanic knowledge, oration, and combat (Radcliffe-Brown, 1964; Henrich et al., 2015). In more sedentary societies, the “Big Man” phenomenon has been observed in places like Melanesia where, in addition to skill, wealth and economic production can elevate individuals’ status resulting in the attraction of many followers (Sahlins, 1963; Henrich et al., 2015). In turn, followers create more wealth as the “Big Man” influences them and organizes them toward more efficient economic production, creating a surplus which he will share. In this way, the prestige dynamic creates a positive feedback loop of followers and success. In some cases, prestige may take a self-aggrandizing turn and status can become a tool for exploitation and strategic manipulation of followers, but the main component of prestige is simply the initial ability attract and influence followers in the first place (Henrich et al., 2015). In addition to field observations, experimental laboratory studies of prestige dynamics have found that young children were twice as likely to copy an adult model to whom bystanders were attending to versus an adult whom bystanders ignored (Chudek et al., 2012). Experiments with adults have demonstrated that both prestigious and dominant leaders emerge naturally in previously unacquainted groups; both types of leaders were judged as influential, but were ascribed different qualities, such as respected and liked (prestigious leader), or “bossy and pushy” and not well-liked (dominant leader) (Cheng et al., 2013).

The different hierarchical systems of dominance and prestige require different evolutionary explanations. For dominance, it is relatively simple: it arises in a context of competition for resources, where there is variation among group members in their ability to acquire resources in the presence of others (Hawley, 1999). Because individuals are typically more interested in their own success than that of their groupmates, competition arises over resources and reproductive opportunities. This leads to a case where the larger, stronger, or, in some species, better connected individuals use their strength (collectively or individually) to increase their access to such resources. For example, subordinate male chimpanzees may invest in grooming other males over time in order to build bonds with them that will pay off in the form of coalitionary support when they fight the current alpha (Foster et al., 2009). This benefits the strong, at the expense of the weak, but by definition the weak are unable to do much about this and so (provided there are no changes to the relative strengths of individuals or alliances, such as a dominant getting ill) stable dominance hierarchies can emerge. Prestige defies such explanations though, because, as discussed above, the individuals at the top of the hierarchy (i.e., prestigious individuals) typically behave with kindness and generosity toward subordinates, and, moreover, rather than taking advantage of this, subordinates respond positively, showing deference.

Unlike dominance, prestige is unique to our species, and as such its evolutionary explanation must be human-specific too. A current explanation for prestige is that it evolved to solve a new kind of problem that arose alongside the human cultural capacity (Henrich and Gil-White, 2001). Specifically, where there is disparity in knowledge or skill among the members of a group, who should you learn from? In some cases, it may be possible to assess the quality of the information offered by potential models. In such instances we should expect individuals with high quality information to be sought out as demonstrators, and in exchange for this information they may be given preferential access to resources or other benefits, as otherwise there would be little motivation for them to share information (beyond close kin, Fogarty et al., 2011). However, in many cases we can expect it to be difficult and/or costly to accurately assess the quality of information offered by different potential models. It would be generally time-consuming to try out each model’s method, and if resources are scarce and/or in themselves costly to obtain, such as the wood and other materials required to build a kayak, then there wouldn’t be opportunities for trial-and error. In this case an observer might rely on the behavior of their groupmates; observing who other individuals are copying and using that as a heuristic to identify and adopt the most valuable information. At this point, selected individuals are no longer being copied as a direct result of their skill or quality as a model, rather they are being copied on the basis of a social consensus that they are highly skilled and so should be copied. It is in this sense that prestige is socially conferred. But what is given can be taken away and so an individual’s prestige may wax and wane over time. Because prestigious individuals receive benefits from their groupmates they have a vested interest in maintaining their position, and hence, it is argued, they behave prosocially and generously toward other group members such that both parties see the benefit of the prestige hierarchy (Henrich and Gil-White, 2001; Henrich et al., 2015).

The fact that prestige is socially determined, and not based on direct evaluation of model quality, raises the possibility that prestigious individuals may not actually have the highest (or even high) quality information. While this is certainly a possibility, observers likely do have some information about quality and so prestige is unlikely to be entirely divorced from model quality. Nonetheless, prestige does not need to be perfectly directed to be adaptive. As long as it directs social learning more effectively than it would be otherwise, it will increase fitness and so be favored by selection. This selection will shape human psychology to further entrench prestige hierarchies and prestige-biased copying. The outcome of this is that humans have an adaptive, evolved preference to defer to and copy people of high social status or “who seem generally popular,” and this preference yields an improved quality and fidelity of socially-transmitted information (Henrich and Gil-White, 2001). This results in a system where prestigious individuals are continually sought as models and receive many benefits in exchange for their information, such as positions of leadership and access to resources. Thus, prestige systems are not altruistic but rather mutualistic, with low status individuals offering deference in exchange for high quality information. Moreover, this explains why prestige evolved only in humans, since only humans rely on cultural inheritance to the extent that prestige is necessary.

## The Human Autonomic Nervous System (ANS) and the Polyvagal Theory (PVT)

In this section, we outline and review the evidence for the polyvagal theory, a biobehavioral model that relates the functions of the human ANS to behavioral self-regulation and social engagement. We then describe how these behavioral correlates of the human autonomic functioning map onto behaviors which are characteristic of dominance and prestige hierarchies.

Vertebrate nervous systems can be divided into two main parts: the central and peripheral nervous systems (PNSs). The central nervous system (CNS) is situated in the brain and the spinal cord, the latter of which, in large part, carries information to and from the brain. The PNS connects to the CNS and can be further divided into two sub-systems: (i) the somatic nervous system (SNS), which includes nerves that carry motor signals from CNS to skeletal muscles and is associated with the *voluntary* control of these muscles, and (ii) the ANS, which includes nerve connections between internal organs and the brain and is associated with *involuntary* control of bodily processes like breathing, digestion, and heartbeat. The ANS is again divided into two sub-systems: the sympathetic (associated with “fight or flight” responses) and parasympathetic (associated with restoration and growth, or “rest and digest”) systems. Where organs are innervated by both the sympathetic and parasympathetic nervous systems, they have antagonistic influences on each other. For example, pupils dilate via the sympathetic ANS, but they constrict via the parasympathetic ANS.

Freezing is a common fear response across species. Freezing helps to avoid detection by predators and can also prepare an animal for action. It entails slowing of the heart rate and it is regulated by the parasympathetic nervous system. It is not simply a passive state, but rather can be thought of as a parasympathetic “brake” on the motor system, temporarily putting sympathetic systems on hold and preparing the animal for further defense responses (including sympathetically-regulated fight or flight) when this brake is released (Roelofs, 2017). It is important to note that during freeze as well as during many other processes, the sympathetic system does not completely shut down or become inactive, but rather it is selectively dampened by parasympathetic dominance. Because of this, heart rate, for instance, is therefore not always an indicator of arousal, since an animal can slow its heart rate via its parasympathetic system even while its sympathetic system remains active, promoting increased awareness and perception. Nonetheless, freezing can lead to tonic immobility or “playing dead” in the cascade of fear responses (Roelofs, 2017).

Mammals exert parasympathetic control of their hearts every time they breath out: during exhalation, the parasympathetic nervous system slightly slows the heart rate. This naturally occurring variation in heart rate during the breathing cycle is called respiratory sinus arrythmia (RSA). On the in-breath, heart rate increases which circulates blood oxygen throughout the body, and on the out-breath, the heart rate slows and energy is conserved. RSA has been widely and almost exclusively observed in mammals (though for a recent example of RSA in lungfish, see Monteiro et al., 2018). Heart-rate deceleration, both during RSA in mammals and during freeze responses in reptiles and mammals, is controlled by the parasympathetic system via the vagus nerve. When RSA is measured, it is a reliable way to assess the parasympathetic innervation of the heart mediated by the vagus nerve (Philips and Donofrio, 2009).

In humans, the vagus is the 10^*th*^ cranial nerve. It is a bi-directional mixed sensory/motor nerve; that is, it sends motor information from brain to organs, as well as sensory information from organs to brain. The many connections of the vagus form a family of neural pathways. The name, “vagus,” is derived from Latin, meaning “to wander” and this name is reflected in the structure of the nerve: it has the longest and widest distribution of any nerve in the body, and provides motor fibers to all organs (except adrenal glands) ranging from the neck to the abdomen, including the throat, ears, heart, lungs, and digestive tract. Indeed, the vagus comprises 75% of all parasympathetic nerve fibers and is the predominant neural effector (Czura et al., 2007).

Porges (2011) proposes that mammals have evolved not one, but two branches of the vagus, a state he refers to as “polyvagal.” The branches can readily be identified in mammalian anatomy, develop separately from each other, and are argued to have separate evolutionary origins. While, for the most part, each branch innervates different organs and muscles, they both exert control over the heart, and do so to two adaptive ends: (1) the first branch exerts control over the heart for metabolic conservation (this function is shared with reptiles) and (2) the second branch exerts control over the heart in the service of social behavior (this function is exclusive to mammals). The two different mammalian vagal branches originate in multiple nuclei of the brainstem: (1) the dorsal motor nucleus of the vagus (DVN), and (2) the nucleus ambiguus (NA), a large group of motor neurons (see Taylor et al., 1999 for an extensive review).

Freeze responses in mammals and reptiles follow the phylogenetically older pathway of the dorsal vagal complex (DVC) originating in the DVN. Signals sent along this pathway slow the heart rate during a freeze response. The dorsal branch of the vagus also innervates organs below the diaphragm, serving digestive function; specifically, stimulating increased secretion and motility in the gastrointestinal tract (“Cranial Nerves: Visceral Motor,” Patestas and Gartner, 2016). In mammals, the newer vagal branch originating in the nucleus ambiguus (NA) also innervates the heart, as well as several organs and muscles above the diaphragm. Specifically, it supplies fibers motor neurons to the larynx (vocalizations and breathing), soft palette (swallowing and breathing), uvula (gag reflex), and pharynx (vocalizations and breathing), as well as the ear canal (sound transmission), tragus (sound direction/location), and auricle (this visible part of the ear; amplification of sounds). Near this auricular region, it also joins up with the facial nerve (cranial nerve XII). All of these muscles innervated by the supradiaphragmatic vagus are involved in human communication and sociality. The polyvagal theory proposes that the addition of this vagal nucleus in the NA, with its connections to the heart as well as muscles in the neck and face, represents an evolutionary change in the ANS to facilitate social engagement (Porges, 2011). In effect, this newer vagal pathway can be considered to act as a parasympathetic “brake” on hostile or avoidant sympathetic impulses during social interactions.

As mammals with complex social lives, this “brake” is useful to humans because they must be able to balance the activity of their sympathetic and parasympathetic ANS in the service of emotional and behavioral regulation in response to social inputs. An example of this is eye contact, which can be a cue of threat or signal of engagement during communication. Non-human primates engage in eye contact in both agonistic and affiliative behaviors. Among rhesus macaques, staring frequently co-occurs with threatening vocalizations toward conspecifics (Partan, 2002). Different species also show divergent preferences for the amount of eye contact in general: bonobos, who are more affiliative and less aggressive than chimpanzees, show more of a preference for facial characteristics (eyes, mouth) of conspecifics versus other body parts compared with chimpanzees (Kano et al., 2015). In keeping with this, studies with human adults find that attention to eyes increased in both affiliative and threatening situations (Kleinke, 1986; Emery, 2000). Similarly, preferential attention to eyes emerges early in human development, with infants preferentially looking at faces that engage them in mutual gaze (Farroni et al., 2002). A parasympathetic “brake” when eye contact is made would be necessary for facilitating positive social interactions. When parasympathetic activity is lacking, people may overinterpret the threat of eye contact and/or they may avert their gaze. Among human adults, for example, gaze fear and avoidance are common among those with social anxiety disorder, a disorder which involves the intense anxiety or fear of being judged, negatively evaluated, or rejected in a social situation (Social Anxiety Disorder, 2010–2018; Schneier et al., 2011; Weeks et al., 2013).

The ability to distinguish human voices from other sounds is also important in human social interactions. The auricular branch of the vagus can trigger an involuntary contraction of the middle-ear muscles, known as the acoustic reflex. The acoustic reflex occurs immediately before a person starts to speak or when other people are speaking and serves to selectively amplify perception of human vocalizations by decreasing the sensitivity of the ear to low-frequencies. Table 1 shows the different ANS responses which have been described so far, along with examples of corresponding muscles/organs and behaviors resulting from these responses.

**TABLE 1:** Bio-behavioral correlates of autonomic neurological structures.

**ANS response**	**System**	**Associated organs/muscles**	**Behavior/function**
“Freeze”	Parasympathetic: “vegatative” vagus via the dorsal vagal complex (DVX)	Heart-rate deceleration (neurogenic bradycardia)	Metabolic conservation Immobilization, potential tonic immobility (death- feigning)

“Flight” or “Fight”	Sympathetic	Heart-rate acceleration Eyes, etc.	Mobilization: fleeing away or aggression. Pupil dilation, etc.

Social engagement system and “immobilization without fear”	Parasympathetic: via the evolutionary newer “ventral vagal complex (VVC)”	Heart-rate deceleration Middle ear muscles	Increased vagal tone measured by RSA (respiratory sinus arrythmia) Selective amplification of human vocalizations over other sounds; listening
		Striated muscles of the face and neck	Facial expressions, head turning
			“Emotional” cueing of the eyes, eye contact
			Head turning and tilting
		Pharynx and larynx	Prosody in speech
		General social engagement system	Quiescient social states: conception, nursing, physical proximity to others, etc.

*Modified from Lenfesty and Fikes (2017b) after Porges (2011).*

One of the main ways the vagus mediates social behavior is by promoting calm states, and this is done through control of the heart. Healthy individuals exhibit parasympathetic control of their heart via the vagus during breathing. This phenomenon is known as RSA, the natural covariation between respiration and heart rate. RSA is believed to occur in order to increase the efficiency of the exchange of oxygen and carbon dioxide between the alveoli (via ventilation) and pulmonary capillaries (via perfusion/blood circulation) (Yasuma and Hayano, 2004). RSA is an index of parasympathetic – specifically, vagal– control of the heart. Under resting conditions, healthy individuals exhibit periodic variation in the length of time between each beat of their heart (R-R or inter-beat intervals measured during an electroencephalogram or ECG) during breathing (Shaffer and Ginsberg, 2017). The measurement of inter-beat intervals allows quantification of heart-rate *variability* (HRV). In general, exhibiting HRV during a resting state is a sign of good vagal tone and suggests an individual should be able to recover more quickly (i.e., slow down their heart rate) from a stressful stimulus than someone who has low or poor vagal tone. In other words, good vagal tone as HRV equates to increased parasympathetic activity and the ability of the individual to quickly “switch gears.”

Research using RSA methods have shown that healthy vagal tone is associated with human social engagement and self-regulation (Geisler et al., 2013), including the capacity for sympathy among children (Taylor et al., 2015) and compassionate responses toward others’ suffering (Stellar et al., 2015). Poor vagal tone is associated with health problems including pre-term, high-risk infants (Shinya et al., 2016), and anxiety disorders in adults (Chalmers et al., 2014), including military combat veterans with post-traumatic stress disorder (PTSD) (Jovanovic et al., 2009) and victims of childhood abuse and neglect (Dale et al., 2009). The causal relationship between RSA and behavior is complicated though, and socialization has been found to improve vagal tone among people who are depressed (Schwerdtfeger and Friedrich-Mai, 2009).

The polyvagal theory argues that the mammalian ANS has evolved two vagal pathways which, when activated, have different behavioral outcomes. The first pathway functions to process threat and fear and elicits a “freeze” defense response which slows of the heart via the DVC, a pathway that mammals share with reptiles. The second parasympathetic pathway also regulates the heart via the vagus, but in addition innervates muscles in the head and neck. This pathway functions to support calm states and human prosocial interactions, including communication.

The stimuli and behaviors which correspond with the activation of the two vagal branches of the mammalian ANS map on well to what we observe in dominance and prestige hierarchies. We expect that the fear and threat that dominant individuals wield (including fear of and threat of punishment, supernatural or otherwise) are processed by receivers via the evolutionarily older DVC. This prepares receivers to submit and do nothing (shut down or freeze) or prepare to take action (flee or fight the dominant). In contrast, we expect that prestige-biased transmission involves the activations of newer vagal pathways which enable individuals to calmly engage with and pay attention to the prestige figure in order to learn from them by listening, communicating, observing, and making eye contact with them without either party inferring a threat or challenge. And while many non-human mammals, including primates, exhibit the kind of social engagement or affiliative behaviors expected from the phylogenetically newer vagal pathways – such as nursing, grooming, bonding, huddling, non-aggressive proximity or physical contacts – they lack prestige, so there is no synergy between these autonomic processes and prestige-biased social learning. We expect that in humans, healthy functioning of the newer ANS pathways supports and interacts with prestige dynamics to produce a bulk of cooperative behaviors that cannot be produced by fear-responses in dominance hierarchies. We will now turn our focus toward how prestige and dominance hierarchies and their complementary autonomic pathways can (or cannot) support the evolution of prosocial behaviors, particularly in the context of religion.

## Two Pathways to Cooperation

Before addressing the possible range of cooperative behaviors possible for humans given our evolved physiology (ANS) and evolved social structures (dominance and prestige), we will briefly summarize just how prosocial we are as a species. If domestication is the process by which other species came to live alongside our own, then human self-domestication is, in part, the process by which humans became remarkably more collaborative with conspecifics. Indeed, compared to any other vertebrate species, we are far more cooperative. Humans help others through alloparenting (Hrdy, 2011; Bentley and Mace, 2012), caring for the sick and needy, and supplying and/or building public goods. Human cooperative relationships are frequently based on reciprocity in which behaviors are tracked and exchanges are delayed (Trivers, 1971; van Veelen et al., 2012). Humans are “ultrasocial,” but this ultra-sociality is puzzling in that it is not explained through genetic relatedness (Mathew, 2015). Humans frequently interact with and cooperate with non-kin, and, compared with other species, this is highly unusual if not completely unique (but see West et al., 2011, for criticism). Given this, the scope and evolution of human cooperation has been a major focus of study for several decades. Here we will explore the potential roles of dominance and prestige, as well as cultural evolution, in this process.

The ability of dominance to foster cooperation must be limited. Indeed, if it were not then it would be hard to explain why cooperation like that of humans is so rare while dominance hierarchies are widespread across species. Nonetheless it is important to understand why this is the case. First let us consider how dominance might support cooperation: when the dominant individual demands it of their subordinates, and punishes those who disobey. If a low-ranking individual defects, they may be punished by higher-ups, and so cooperation may be somewhat stable through coercion. But what if it is a high-ranking individual that defects? Indeed, there is little to stop dominant, selfish individuals exploiting cooperators while not being cooperative themselves. As such, the kind of cooperation fostered by a dominance hierarchy is very different to that observed in human behavior. This is indeed what we observe in many non-human species as discussed above; dominant individuals force subordinates to behave “cooperatively” (i.e., in the interest of the dominant). So, subordinates behave cooperatively, but dominants are selfish.

However, even the ability of dominants to enforce cooperative behavior among subordinates may itself be limited because dominant individuals can enforce cooperative behavior only when they are present. When absent, a leader would have to rely on a loyal network of minions to monitor and enforce behavior of subordinates. This, however, creates the problem of second-order free-riders who could possibly defect on carrying out punishment. Given this, as group sizes grow larger, it would become impossible for a leader to monitor all behavior and so the attempts of dominant individuals to enforce cooperation amongst subordinates cannot be entirely successful.

Dominance as a mechanism for sustained cooperation is thus limited. One solution to the problem of both first- and second- order free-riders in large groups is punishment from moralizing, omniscient, “Big” gods (Norenzayan, 2013; Norenzayan et al., 2016; Purzycki et al., 2016). Under the Big Gods hypothesis, supernatural agents who are believed to monitor behavior and punish transgressions are an effective way of supporting cooperation in large groups. Johnson (2015) makes a similar argument that belief in supernatural punishment can support cooperation, but that it primarily provides individual-level fitness benefits: since individual selfish behavior can be costly, belief in supernatural punishment can inhibit individuals’ selfish impulses and thus save them the cost of being punished by other people (so long as punishment from others is present and efficacious in that individual’s social network). Regardless of the level(s) of selection upon which supernatural punishment operates, the deity is effectively taking the place of the dominant individual. Fear is key to these hypotheses. Believers must actually fear the god(s) and believe that they will be punished if they transgress a norm. In this way, fear of punishment is supposed to curb anti-social behavior and gods are like dominant leaders: they wield their influence through intimidation and force, and followers submit to them out of fear. Note that this solves the two problems with dominance supporting cooperation: (1) the deity is assumed to be omniscient and so the issue of monitoring disappears, and (2) the deity is not perceived to be dependent on humans and therefore has no incentive to be tempted into defection because there are no benefits for the deity to reap. Of course, in human societies specific individuals are often assigned the role as being a spokesperson for the deity and so such conflicts of interest will likely re-emerge.

While supernatural punishment accounts of the evolution of cooperation are viable, here we present an alternative that relies on systems of prestige instead of dominance. As already discussed, the existence of prestige-biased learning implies that followers copy traits from prestigious leaders; if in addition to being skillful/knowledgeable prestigious leaders are also cooperative (as they are often described as being), and if followers imitate more of the leaders’ traits than just the target trait (i.e., skill, knowledge), cooperativity could spread through the population. Indeed, in a set of culture-gene coevolutionary models, Henrich et al. (2015) found exactly this: that cooperation can be stable even in large groups when cooperativity is a cultural trait that is learned from prestigious leaders and passed on to future generations by followers.

At this point we will make the same step as proponents of supernatural punishment theories of cooperation by invoking gods of our own: while fear of deities and supernatural agents (and even impersonal moralizing forces like karma) may be widespread across cultures, gods and supernatural agents are also revered, admired, adored, respected, and loved. In short, Gods and supernatural agents are prestigious as well as dominant. Rather than using fear of supernatural punishment to promote cooperative behaviors, prestigious gods/supernatural agents tap into human psychology which is biased toward imitating prestigious individuals to promote cooperation without the need for threatening punishment. Indeed, the spread of cooperation via prestige-biased transmission may not even rely on close monitoring of group members because part of human prestige psychology is an active desire to be like prestigious individuals at all times, and not just in their presence.

In support of a role for prestigious gods, it has been previously argued that fear, and fear of punishment specifically, alone cannot account for the range of observed prosocial behaviors in religions. Lenfesty and Schloss (2015) have encouraged scholars to make a distinction between the mechanisms that simply make people less likely to violate prevailing behavioral norms (i.e., supernatural punishment), and what makes them “nice.” Johnson and Cohen (2016) have similarly argued that “refraining from doing bad” is not the same as “doing good.” Lenfesty and Fikes (2017a, b), after Porges (2011), have proposed that these two categories of prosocial behaviors arise from separate neurophysiological systems. Critically, experiments have shown that different types of prosocial behaviors result from different perceptions of god. For example, Johnson et al. (2013) found that among both Catholics and non-Catholic Christians, people who had predominantly authoritarian concepts of God were more aggressive while those with a predominantly benevolent concept of God were inclined toward volunteerism and showed greater willingness to aid religious out-groups. In another study by Johnson et al. (2013), participants were given reminders of a either a benevolent or authoritarian god; the former type increased the willingness to forgive, while the latter type increased aggression and decreased forgiveness, as well-decreased the willingness to conserve water, volunteer, or aid religious out-groups. While Johnson et al. (2013) did not explicitly use the words “dominance” or “prestige” in their Authoritarian/Benevolent God scale, the majority of their items overlap with experimental, ethnographic, and historical descriptions of traits of dominant and prestigious leaders.

As previously discussed, feeling fear frequently results in a physiological and behavioral “freeze” response, and while freezing may inhibit some antisocial behaviors, it cannot lead to other kinds of social engagement behaviors like helping and caregiving. If gods are predominantly fearmongers, prosocial behaviors will be limited to what only fear can achieve. In order to facilitate approach-driven prosocial behaviors, the environment would have to provide more positive cues that could in turn activate parasympathetically-mediated social engagement behaviors.

Note that our argument is not that dominant deities cannot promote prosocial behaviors, or that proponents of supernatural punishment theories of religion are wrong. Rather, we suggest that there is a second means by which gods can be used to promote cooperation. Cooperation can be demanded by dominant gods who threaten punishment if disobeyed, or it can be requested or inspired by prestigious gods who offer a chance to share in their benevolence if acquiesced to. These should not be viewed as mutually exclusive options, and a carrot-and-stick mix of prestige and dominance may well be the norm. Moreover, different groups within the same religion may emphasize and focus on either the fearful or benevolent aspects of a given deity.

## Cases of Prestige-Driven Dynamics in Religious History

We have now outlined the core of our theory: that prosocial, prestigious gods can tap into human psychology to promote cooperative behaviors in a way that dominant gods cannot because the two types of deities activate different neurophysiological systems. In this final section we will review historical cases that show the effects of prestigious gods in action in order to provide evidence that prestigious gods have been an important part of the evolution of human behavior.

Prestige dynamics can be observed in many religions or spiritual groups where highly revered central figures act as teachers and guides (as opposed to domineering aggressors) who disperse their knowledge and skills to followers who are inclined to imitate their ways. All branches of Buddhism are centered around Gautama Buddha’s life and teachings, mainly the Four Noble Truths (i.e., the truth of suffering, the truth of the cause of suffering, the truth of the end of suffering, and the truth of the path that leads to the end of suffering) and the Noble Eightfold Path (i.e., right understanding, right thought, right speech, right action, right livelihood, right effort, right mindfulness, and right concentration). Interestingly, the story of Siddhartha Gautama (a.k.a. the Buddha) involves him renouncing his position within a class of ruling elites (an already prestigious position) and giving up all the wealth associated with it and becoming a monk and traveling teacher. In Hinduism, gurus are literally “ignorance dispellers” (Sanskrit: *gu*, “ignorance,” *ru* “dispeller”): highly revered teachers who serve as models and guide students not only in the development of spiritual knowledge, but also the arts (e.g., music and dancing) (Mlecko, 1982). Islam centers around the teachings of the prophets (Noah, Abraham, Moses, Jesus, and most importantly, Muhammad), who model ideal human behavior and are revered as messengers sent by God.

Modern Anglican bishop and theologian Wright (1999) asserts that, historically, Jesus stood apart from the other religious leaders with regard to one event in particular: his alleged resurrection. Historically, the Christian worldview has been centered around escaping death and obtaining eternal life. Jesus may have acquired prestige because his followers believed he was the only person ever to have escaped death through his resurrection, an immensely valuable skill they hoped to share in by following his teachings. To the Jews, death meant a denial of God’s good created order and the Hebrew prophets of the Torah predicted a time when God would restore this order (Wright, 1999). The Christians saw Jesus as the restoration of the world order and believed that by having faith in him and a commitment to his values they would receive the same ability to escape death, that is, the promise of eternal life. A cultural evolutionary reading of the Biblical texts portrays Jesus receiving deference from his followers in exchange for his fitness-relevant expertise in the domain of social norms (e.g., the behaviors required to live a moral life, how to navigate life’s challenges); the added expertise on how to overcome morbidity and obtain eternal life likely made him even more sought after.

Thus, the role of religious figures as knowledge-bringers (a key part of prestige systems) seems widespread. However, another part of our hypothesis is that prestigious, religious figures specifically advance prosocial behaviors in their flock. While all of the world’s major religions and their leaders explicitly teach about almsgiving, charity, and generosity, we focus on here on early Christianity as an example of prosocial behavior emerging from a prestige hierarchy. In the New Testament, Jesus is portrayed as having a group of dedicated followers (i.e., the disciples) who follow him closely and receive direct teachings from him. In artwork depicting Jesus delivering the Sermon on the Mount – a moralistic message and guide for living, including the commandment to love one’s neighbors and enemies as well as give to the needy – he is portrayed as sitting or standing only slightly above his disciples and the crowd, with an open body posture of both arms extended to his sides or one had gently raised. These gestures fit with description of prestige displays, which are less expansive than dominance displays (Henrich, 2016).

New Testament texts describing the dynamics between Jesus and the disciples also map onto ethnographic and psychological descriptions of prestige dynamics, including the leader-follower dynamics of “Big Men” societies (Henrich et al., 2015), where highly skilled individuals command respect and exert influence over a group of followers. Like many other leaders, the character of Jesus portrayed in the New Testament appears to be a charismatic and highly skilled orator who had the purported power to perform miracles (i.e., heal the sick, feed the masses), which were received as acts of generosity.

A closer look at the rapid rise of Christianity in its first five centuries reveals how Jesus himself as well as the disciples who went on to preach his gospel (i.e., Paul) were successful not only in accumulating followers on his behalf and advocating for prosociality, but also in promoting prosocial behaviors amongst believers, which in turn further drove the growth of the fledgling religion. In a 260-year period, Christianity rapidly expanded from an obscure Messianic cult movement in the far edge of the Eastern Roman empire to an estimated size of 5–7.5 million members (Stark, 1996). Sociologist Rodney Stark attributes the success of Christianity to several key factors, including the highly prosocial response of Christians to two severe plagues that ravaged the empire between the 1^*st*^–5^*th*^ centuries AD. The Antonine Plague swept through Roman Empire from 165 to 180, resulting in the loss of an estimated quarter to a third of the entire empire’s population during the first plague (Boak, 1947; Russell, 1958; Gilliam, 1961; McNeill, 1976). By the end of the second plague, the Plague of Cyprian from 251 to 266, classical society was severely “disrupted and demoralized” (Stark, 1996, p. 74). During this time, Pagan leaders and government officials offered no assistance or care for the sick, while Christianity – still a minor but increasingly growing movement – did.

There is little historical textual evidence to suggest that fear of God was a primary motivator in the nursing practices of Christians in the first five centuries AD. This is because even though Christians faced severe losses, they viewed the plagues as trials of their faith and not as direct punishment from God. Eusebius’ history of the early church includes an Easter letter from Dionysius, the bishop of Alexandria, during the plague in 250, in which he writes that “heathens” faced even greater losses because of how terrifying it was to them (p. 240). Dionysisus contrasts the behavior of the two groups:

Most of our brethren showed love and loyalty in not sparing themselves while helping one another, tending to the sick with no thought of danger and gladly departing this life with them after becoming infected with their disease. Many who nursed others to health died themselves, thus transferring their death to themselves. The best of our own brothers lost their lives in this way – some presbyters, deacons, and laymen – a form of death based on strong faith and piety that seems in every way equal to martyrdom. They would also take up the bodies of the saints, close their eyes, shut their mouths, and carry them on their shoulders. They would embrace them, wash and dress them in burial clothes, and soon receive the same services themselves.

The heathen were the exact opposite. They pushed away those with the first signs of the disease and fled from their dearest. They even threw them half dead into the roads and treated unburied corpses like refuse in hope of avoiding the plague of death, which, for all their efforts, was difficult to escape.

(Maier, 1999, p. 240)

Although it could be argued that Dionysius had a biased view of Christian charity, Harper (2016) makes the point that it would be difficult to exaggerate or fabricate an experience that his audience was experiencing first hand.

There are also pagan accounts of how the responses of the two groups differed. The Christian movement continued to grow after the plagues and by 313 it was enough of a force that the emperor Constantine finally made it a legal religion in the empire with his Edict of Milan. 100 years after the second plague in 362, the Emperor Julian wrote to Arsacius, the high pagan priest of Galatia, with some instructions on how to rescue the waning pagan religion which was severely lacking in the area of public service. Julian ordered Arsacius to basically force other priests to copy what the Christians were doing, and punish them if they did not conform to his orders:

The Hellenic religion does not yet prosper as I desire…. why do we not observe that it is their benevolence to strangers, their care for the graves of the dead and the pretended holiness of their lives that have done most to increase atheism [i.e., Christianity]? I believe that we ought really and truly to practice every one of these virtues. And it is not enough for you alone to practise them, but so must all the priests in Galatia, without exception. Either shame or persuade them into righteousness or else remove them from their priestly office…

(Wright, 1913)

Julian saw the need to compete with Christians, who were helping both their fellow Christians as well as pagans. Julian promised to send Arsacius large amounts of food and wine as a way to entice “strangers and beggars,” especially, into the pagan religion. But he also used shame to motivate the cooperation of his subordinate, writing:

For it is disgraceful that, when no Jew ever has to beg, and the impious Galilaeans [i.e., the Christians] support not only their own poor but ours as well, all men see that our people lack aid from us. Teach those of the Hellenic faith to contribute to public service of this sort…let us not, [allow] others to outdo us in good works.

(Wright, 1913)

Interestingly, Julian’s letter represents a dominant strategy to cooperation: even if the end goal was providing social services, this should be done through use shame and threat of punishment to the “management,” i.e., the priests carrying out the services.

Martyrs were also an important source of inspiration to Christian prosocial behavior and an extreme example of what early Christians were willing to give up to benefit their group. Eusebius’ *Church History* reports several stories of early Christians being beheaded, burned alive, and tortured. These accounts portray the martyrs as recalcitrant to the Romans even while being tortured. Galen, the famous Greek physician to the imperial court of Rome, was present during the Antonine Plague. Of the resilience and virtue of the Christians, he wrote,

For their contempt of death is patent to us every day…and they also number individuals who, in self-discipline and self-control in matters of food and drink, and in their keen pursuit of justice, have attained a pitch not inferior to that of genuine philosophers.

(Walzer, 1949, p. 15)

Martyrdom likely served two important and related functions during this time of early Christianity. First, it reinforced the Christian belief that death (by martyrdom or disease) shouldn’t be feared because they believed that eternal life was possible. For reasons discussed previously, the strengthening of this belief may have damped the amount or degree of fearful states that would have prevented social engagement (helping, communication) among Christians.

Second, martyrdom in early Christianity maps well onto the cultural evolutionary theory that such displays or “CREDS” (CRedibility Enhancing Displays) operate through an evolved social-learning psychology where learners use models’ behavior to determine how much to commit to a belief (Henrich, 2009); in this case, the models’ behavior is very costly so their credibility is high which strengthens the observers’ motivation to buy into the ideology. Henrich’s (2009) analytic model shows that the stable presence of CREDS in groups can increase the success and competitiveness of a group by increasing cooperation. This is possible because the CRED (a) is an altruistic act which provides direct group benefits, (b) is an act of punishment that penalizes non-cooperators, and/or (c) delivers no immediate *direct* benefits to the group but elevates and stabilizes an ideology that favors other group-beneficial behaviors (Henrich, 2009). In the case of early Christianity, type (a) CREDS include helping/nursing fellow Christians (plus any pagans who would later convert to Christianity because they were cared for) and type (b) CREDS include martyrdom which would presumably lead to more prosocial behaviors of the (c) type.

These beliefs and behaviors are nested within a religious prestige hierarchy where followers are also motivated to attend to a central figures’ (i.e., Jesus’) expertise on how to obtain eternal life. The positive feedback loop effect of prestige, where an increase in the number of followers increases the prestige of the prestigious person, which in turn increases the figures’ sphere of influence, seems to have been a major part of Christianity’s success. This, combined with the CREDS of Jesus himself like healing disease and providing food to the masses, as well as the crucifixion (which was interpreted as an extreme sacrifice, i.e., dying for the sins of humankind), would have increased prosociality within early Christianity. Finally, specifically Christian beliefs that functioned to dampen fear responses in the body would have benefited Christians’ social relationships by allowing them to maintain calm autonomic bodily states.

Since the time of its beginnings under the Roman Empire, Christianity has become the most widely practiced religion in the world. Although its practices and theology have widely varied, as seen through the rise, fall, and maintenance of hundreds of denominational sub-groups, examples of prestige concepts are prominent it its modern form. For example, Hillsong Church – a Christian megachurch in Australia with weekly services of over 43,000 with an additional 1.7 million people in other parts of the world linked to a livestream of its service (Hillsong Church Annual Report, 2018) – is a major producer of contemporary Christian music with lyrics that promote a prestige-concept of God and Jesus. Hillsong’s worship music-centered YouTube channel has 4.7 million subscribers (as of October, 2019); the lyrics of one popular song, “To Be Like You,” say: “I will walk/In Your ways/Love Your word/Seek Your face/My reward/My sole pursuit/To know You more/To be like You/Jesus/All I want is to be like You.” The lyrics of another popular worship song by River Valley Worship (YouTube channel with 9.4k subscibers as of October, 2019), “Wanna Be Like You,” similarly say: “Come and change my heart/show me who You are/I wanna be like You, I wanna be like You/Take my heart, my soul, I give You control/I wanna be like You, I wanna be like You, Jesus.” Grammy-award winning and top Billboard chart-achieving gospel singer Tasha Cobbs Leonard’s song, “By your spirit,” says: “Not by might/Not by power/By your spirit God/Send your spirit God/You called us out/Out of the darkness/Into your love/Into your light/Grace upon grace/Beauty for ashes/You come to us/We come alive/We stand in awe of you.” These are just a few examples of many contemporary worship songs that emphasize the imitation of the traits of supernatural prestigious figures.

Regarding how prestige hierarchies in religion can foster healthy autonomic functioning, and therefore lead to prosocial behaviors, Luhrmann (2012) describes in her ethnography of the popular Vineyard Church how members its members’ emotional states and behaviors change as a result of conceptualizing a benevolent and loving god. She writes, “People are told that they are safe and loved…. When people feel lovable, they are less likely to interpret a curt tone as an insult…the social life of evangelical churches is rich in specific emotional practices…these emotional practices create powerful feelings…they lead the congregant to want to change and practice the change, [and practice] the experience of being loved by God.” We expect that when cultural narratives of benevolent and prestigious supernatural agents are reinforced through collective rituals such as singing, prayer, reading of texts, and listening to sermons, this can create safe environments where positive emotions and behaviors – like the desire to be like a help others – can, as a result of calm autonomic states, thrive.

## Conclusion

We have described how the phylogenetically older vagal systems that humans share with animals operate to process fear (threats), as well as how dominance hierarchies are characterized by fear and intimidation. Given that humans, like all mammals, have phylogenetically newer vagal adaptations for social engagement which suppress fear responses, and that human social life is characterized not only by dominance but also by prestige dynamics, we hypothesize that such newer vagal pathways function in part to support this alternative social hierarchy of prestige. We therefore expect that cultural traits like prosocial/altruistic religious ideas and practices will capitalize on these systems and spread via cultural evolutionary processes.

The evolution of a prestige psychology in humans opens up a new means by which religions can shape human behavior. Prestige psychology means that humans are predisposed to show deference toward individuals that display the key markers of prestige: generosity and benevolence, as well as being deferred to by other individuals. This applies to deities and supernatural agents, as well as flesh-and-blood individuals: a divine being that displays these traits can tap into human prestige psychology and prompt deference and imitation. As such, a deity or supernatural agent that not only displays prosocial tendencies, but also actively encourages the cultivation of prosocial dispositions among their followers can promote cooperation in a way that a punishing deity cannot. By promoting prosocial behaviors such deities/supernatural agents can enhance the fitness of the group of their followers, allowing their beliefs to spread through, for instance, cultural group selection. This process can be seen at work in the cultural dynamics of religions, including early and modern Christianity, which placed great emphasis on Jesus as a prestigious rather than dominant figure. Thus, religions need not be based on a vengeful, punishing God to promote group-beneficial behaviors. Instead there is an alternative pathway: benevolent, generous and prestigious gods can promote prosociality by tapping into our prestige psychology that is primed to defer to, and copy, such figures.

## Author Contributions

HL conceived the idea. HL and TM wrote the manuscript.

## Conflict of Interest

The authors declare that the research was conducted in the absence of any commercial or financial relationships that could be construed as a potential conflict of interest.

## Footnotes

1 We are using “awe” in its contemporary positive sense, not the older sense in which includes notions of fear and dread. The latter definition is now considered obsolete by The Oxford English Dictionary (OED), and the positive definition has been in use for the last 700 years: “From its use in reference to the Divine Being this (i.e., immediate and active fear; terror, dread) passes gradually into: dread mingled with veneration, reverential or respectful fear; the attitude of a mind subdued to profound reverence in the presence of supreme authority, moral greatness or sublimity, or mysterious sacredness.” Furthermore, the phrase “to stand in awe of” has transformed over time, from meaning “to be greatly afraid of” to “entertain a profound reverence for” (Awe, 2019).
